# Application of the Neonatal Behavioral Assessment Scale to Evaluate the Neurobehavior of Preterm Neonates

**DOI:** 10.3390/brainsci11101285

**Published:** 2021-09-28

**Authors:** Roksana Malak, Brittany Fechner, Dorota Sikorska, Marta Rosołek, Ewa Mojs, Włodzimierz Samborski, Ewa Baum

**Affiliations:** 1 Department and Clinic of Rheumatology, Rehabilitation, and Internal Medicine, Poznań University of Medical Sciences, 61-545 Poznań, Poland; blemus001@gmail.com (B.F.); dorotasikorska@ump.edu.pl (D.S.); wlodzimierz.samborski@ump.edu.pl (W.S.); 2 Department of Rehabilitation, Poznań University of Medical Sciences, 61-545 Poznań, Poland; marta_rosolek@o2.pl; 3 Department of Clinical Psychology, Poznań University of Medical Sciences, 60-812 Poznań, Poland; ewa_mojs@poczta.onet.pl; 4 Department of Social and Human Sciences, Poznań University of Medical Sciences, 60-806 Poznań, Poland; ebaum@ump.edu.pl

**Keywords:** neurobehavior, NBAS, prematurity, neonates, early rehabilitation

## Abstract

Background: The neonatal behavioral assessment scale (NBAS) was primarily developed to aid in the assessment of full-term neonates. The aim of this study was to detect if the NBAS was also valuable in the assessment of preterm neonates. Materials and Methods: We assessed 112 infants at a neonatal unit using the NBAS, 4th edition. The inclusion criteria included an oxygen saturation level between 88–95% and a heartrate of 100–205 beats per minute. Infant neurobehavior was assessed using the NBAS. Results: For full-term and preterm neonates, we observed that the NBAS enabled us to assess both groups of infants and gave relevant information pertaining to them. We found a significant correlation between the average week of gestation and response to touch, sensory input, peak of excitement, cost of attention, hand-to-mouth, and quality of alertness. Conclusions: The NBAS is a valuable scale for evaluating the neurobehavior of preterm neonates. The week of gestation at birth affects certain aspects of neurobehavior, such as response to sensory input, putting hand to mouth, peak of excitement, and cost of attention. The NBAS as an individually structured assessment may help in planning for early rehabilitation and intervention for this vulnerable population.

## 1. Introduction

The assessment of the neurobehavior of a neonate helps guide a practitioner in developing appropriate treatment and intervention. The examination of high-risk patients should take place as soon as possible. An assessment should provide objective knowledge of an infant’s behavior observed during the clinician-patient interaction to allow for the evaluation and prediction of neurodevelopmental outcomes. This can then be used to plan for the early rehabilitation and intervention of services according to the needs of a neonate [1].

The neonatal behavioral assessment scale (NBAS) is a neurobehavioral assessment used for infants up to 8 weeks postmenstrual age (PMA). Thanks to the NBAS, parents and caregivers can attain knowledge not only about the neurobehavior but also the development of their child. Additionally, parents and caregivers may determine what their baby needs and how they can best communicate with them. The NBAS may help improve caregiver–infant interactions when the relationship between an infant and mother is challenging due to the hospitalization of a preterm neonate [2]. It is for sure a stressful situation for a parent when a child stays in the hospital following birth and delivery and subsequently has to undergo stressful medical examinations [3,4]. Therefore, it would be beneficial to reduce stress for parents in such situations. There are many advantages to utilizing neurobehavioral scales. Such advantages include attaining information about the neurodevelopment of the central nervous system (CNS) and its maturation [5]. Neurobehavioral scales help practitioners in understanding neonates as unique individuals whose behavior can be observed as a meaningful way in which to communicate their needs, abilities, challenges, and preferences to others [1]. Understanding this type of behavior enables us to observe how neonates communicate, which in turn helps caregivers in sufficiently supporting and coregulating a neonate’s development and abilities by ensuring that all other physical needs have been met. Although the NBAS helps us in understanding newborns as unique individuals, by observing their communication, wants, needs, abilities, challenges, and preferences, some of these items may vary between full-term and preterm infants or may be challenging to assess due to an infant’s physiological immaturity. Sometimes, this means that not all items of the NBAS may be assessed if a child presents as too fragile or immature.

As a result, it is more common to apply the NBAS assessment with full-term infants rather than with preterm infants. However, the NBAS ensures a holistic and systematic assessment of clinical conditions for both full- and preterm neonates [6]. Early assessment of neurobehavior is the first step towards determining early intervention needs, especially for preterm neonates since there may be differences found between preterm and full-term children [7,8].

The aim of this study was to determine the areas of the NBAS that may be different between these two groups in reference to week of gestation, as well as items that may be demanding to assess for preterm neonates. Appropriately applying the NBAS allows a practitioner to assess and treat a neonate with dignity and to plan for intervention accordingly, if needed.

## 2. Materials and Methods

We examined 112 infants hospitalized at a neonatal unit. All parents and caregivers provided written informed consent prior to the assessment and examination of their child. The clinician explained the process of the NBAS to all participants in the study. This study was approved by the Institutional Review Board at the Poznań University of Medical Sciences (Bioethics Committee, consent reference number 734/19, Poznań University of Medical Sciences, Poznań, Poland). The inclusion criteria for our study’s participants included a stable condition of the neonate, a peripheral oxygen saturation (SpO_2_) between 88 and 95%, and a resting heartrate between 100–205 beats per minute. The pulse oximetry of each neonate was measured on the sole of his or her foot using a Nellcor pulse oximeter [9,10,11]. If a child showed any instability in their condition, in regard to peripheral oxygen saturation or heartrate on the day of examination, then this was treated as exclusion criteria. Exclusion criteria included a desaturation index of SpO_2_ < 88%, a heart rate lower than 100 or higher than 205 beats per minute, active inflammation, sepsis, reflux, bone replacement, the presence of a tumor, encephalopathy, and lethal birth defects (e.g., Edwards syndrome, Patau syndrome etc.). After considering all inclusion and exclusion criteria for the study’s participants, we included 100 patients in our study. Of the 100 patients included in our study, 93 of these NICU patients were born preterm and 7 were born full-term. Each participant was placed on a flat and comfortable surface in a cot incubator during the assessment.

We assessed the neurobehavior of our study’s participants using the Brazelton neonatal behavioral assessment scale (NBAS), 4th edition [12,13]. We observed and assessed the following items from the NBAS in stable infants: habitation, social interaction, motor system, state regulation and organization, autonomic nervous system (ANS), reflexes, and supplementary items [2]. Due to the fact that the NBAS assessment should only be conducted after taking a special course, our researchers were trained from the NBAS course provided by the Cambridge Brazelton Centre in the United Kingdom. During the administration of the NBAS assessment, a qualified clinician administers as many as 28 behavioral and 18 reflex items, depending on time allocation and an infant’s willingness and ability to engage in the assessment [2]. Responses from the NBAS are recorded on a standardized scoring sheet. The exact procedures for assessing a neonate can be found in the official 4th edition NBAS manual, which was written and described by Berry T. Brazelton and Kevin J. Nugent in 2011. Please refer to Brazelton et al. (2011) to find all procedures and protocols pertaining to the NBAS [13]. Only certified NBAS instructors are permitted to teach and describe how to assess newborns using this scale. All rules and procedures were safely performed for the study’s participants and their parents or guardians.

Statistical analysis was performed using STATISTICA 13.0 software (TIBCO Software, Tulusa, OK, USA). The normality of the data distribution was checked using the Shapiro–Wilk test. As the data obtained did not consistently display a normal distribution, differences between unpaired data were analyzed using the Mann–Whitney U test. Categorical data were analyzed using Pearson’s chi-squared test. The correlation coefficient was determined using Pearson’s correlation coefficient (r). The differences found were considered significant at *p* < 0.05. Regarding statistical analysis, we considered the week of gestation and not only the division between full-term and preterm neonates.

## 3. Results

We examined 100 patients for the purposes of this study: 52% were female and 48% were male. The average birth weight of each infant was 1805 g (±656 g). Of the children included in the study, 14% had a normal birth weight, 54% had a low birth weight, 24% had a very low birth weight, and 8% had an extremely low birth weight. The average number of weeks of gestation for newborns was 32 weeks (±3). We took into consideration whether or not neonates who were born before 37 weeks of gestation were more difficult to assess compared to full-term infants or if any item was not possible to perform with them for the purposes of the NBAS. We found the following statistically significant items in the NBAS: consolability, general irritability, and lability of states between full-term neonates (≥36 week of gestation) and preterm neonates (<36 week of gestation) with *p* < 0.05. When we assessed reflexes, we only found statistical significance in tonic deviation of head and eyes present between full-term and preterm infants (Table 1).

We also considered if any features of the NBAS were significantly different to perform regarding week of gestation. We observed statistically significant differences according to week of gestation in assessing children via the NBAS in a few items (Table 2).

## 4. Discussion

The assessment of the neurobehavior of a neonate helps a clinician understand its needs and therefore apply an appropriate intervention as soon as possible. Assessment should be a standardized component of a neonatal clinical observation [5]. This seems to be especially essential for babies who are born premature. Delivery before 37 weeks of gestation is considered preterm. These infants are often placed in very stressful situations as a result of medical procedures that are important for their health early in life. These organisms must fight for their lives by developing any immature body systems. Observing the neurobehavior of a neonate may provide parents and specialists with information regarding how to effectively communicate with and help a neonate with respect to its needs.

Additional neurobehavior assessment scale that may be useful in every day clinical practice in neonatal units is the Assessment of Preterm Infants’ Behavior (APIB) or the NeoNatal Neurobehavioral Scale (NNNS) [1,5,11,14,15,16]. However, we chose to use the NBAS due to its simplicity and positive approach to working with neonates and their parents or caregivers. The collaboration between a therapist and caregiver in understanding and responding to an infant’s behavior can benefit infant development and caregiver-infant interactions over long periods of time [17]. Although it has been shown that the NBAS is especially valid to use with full-term infants, we have observed that it may also be useful for assessing preterm infants [2,18,19]. We found statistically significant differences when using the NBAS according to weeks of gestation in the following items: consolability, general irritability, tonic deviation of head and eyes, and lability of state. Although the NBAS has primarily been developed for the assessment of full-term infants, other researchers and ourselves have shown that this scale may also be important for the assessment of preterm neonates [14,20,21]. However, if we consider the week of gestation apart from the division before or after the 37th week of gestation, we observed that only a few features depended on the week of gestation such as cost of attention; quality of alertness; peak of excitement; and reaction to sensory input, such as response decrement to tactile stimulation of foot and animate visual and auditory response (Table 2). According to Kelly (2019), neurobehavior in regard to brain structure–function associations generally does not differ between very and moderate-late preterm neonates [22]. Thus, we did not compare our results in the subgroups, in reference to week of gestation.

It should be emphasized that we found statistical significance for a just few NBAS items in the division before and after 37 weeks of gestation: response decrement to tactile stimulation of foot, animate visual and auditory, peak of excitement, cost of attention, hand to mouth, and quality of alertness. We hypothesize that the week of gestation is correlated with features that may be connected with the maturity of the nervous system such as reflexes, response to sensory stimuli, quality of alertness, lability of state, quality of alertness, and cost of attention. Tactile stimuli are very important for the process of embodied reparation [23]. Others neurobehavioral assessments, such as the NNNS, showed that greater gestational age was independently related to greater maturity of movement, better state regulation, and less excitability [24]. Similar to our findings, attention as assessed by the NNNS was correlated with week of gestation [22,25]. Immaturity of the nervous system is an important issue related to both the pathophysiology and clinical care of the preterm neonate since the development of the nervous system is most dynamic in the third trimester of pregnancy [26]. This is important later to the neurobehaviors of a child and their state regulation [27].

Preterm infants tend to demonstrate problems with state regulation [28]. Our study showed that the earlier the week of gestation, the more problems with state regulation that the child had. Premature infants often express difficulties with achieving the entire range of states, for example, a defined state of deep sleep [29]. Preterm infants appear sensitive to most parameters of environmental impingement, and this may lead to hyperresponsiveness [30]. This is due to the fact that higher cortical functions in the brain, especially cortical systems that provide buffering and differential inhibitory controls, have not yet fully developed [30]. According to Als et al. (2020), “The sudden passage to an environment poorly matched to their expectations triggers altered subsystem functioning”, which may increase a neonate’s level of reaction when they reach a peak of excitement, thus making them unavailable to the outside world [13,30]. Irritable neonates can be easily disturbed by environmental stimuli and may be slower to respond to attempts to console them. This is why consolability was more difficult in children who were born preterm. Decreasing environmental stimuli, such as light, noise, and sudden movement, may be helpful in consoling a child [20]. Instead of finding themselves in a safe and well-integrated environment, such as their mother’s womb, preterm infants are often placed in environments and situations poorly matched to their expectations. The typical environment of a neonatal unit is full of various stimuli, which may lead to costly defense behaviors that may lead to increased distortion and disorganization in neonates [30]. This may explain why the younger patients in our research group experienced a higher cost of attention, even during minor physical demands such as opening their eyes [30]. Brazelton describes the “cost of attention” as the amount of energy the infant must expend to maintain an interaction, and depends on the maturity of the infant [29]. Infants born premature often show a greater reluctance to become alert [30]. Preterm infants may have brief periods of alertness. Therefore, the quality of alertness and cost of attention tends to be higher in preterm neonates, as our study showed.

In general, the shorter the period of gestation, the poorer the rate of maturation [27]. We initially predicted that reflexes may be especially demanding for preterm neonates [31]. Absent or abnormal reflexes are an indirect indicator of neurological immaturity in newborn infants [32]. When comparing full-term and preterm neonates, we found the following difference in just one out of all assessed reflexes: tonic deviation of head and eyes (Table 1). Since primitive reflexes are involuntary motor responses that develop in the brainstem as early as the 25th or 26th week of fetal life, this may account for the small number of differences found between our two groups [32,33]. Tonic deviation of head and eyes is a reflex that is the body’s response to gravity, which markedly influences vertical eye position and movements [34]. To test this reflex, the examiner holds the infant upright and slowly rotates them 90 degrees in one direction. In response, the infant’s eyes should turn into the movement of the spin [13]. Abnormality or absence of this response may be a valuable indicator to assess since it may be a very important symptom of a brain function disorder that may go undiagnosed during a routine medical examination. Thanks to the NBAS, neonates may receive an appropriate neurological examination even earlier, such as an electroencephalogram (EEG). The differentiation of this reflex is important to appropriately assess and treat [35,36,37].

Additionally, we observed that the higher the week of gestation the better the results were for assessing sensory stimulation such as touch, visual input, and auditory input. Preterm infants have immature organ systems, including their auditory and visual systems [38]. Research shows that preterm infants require specific sensory stimulation in order to initiate oral feeding and achieve a shorter length of stay at the hospital [39]. Current studies show that preterm birth for neonates negatively affects sensory processing [40,41,42]. Furthermore, a younger gestational age appears as a risk factor for developing sensory processing disorders in preterm infants [40]. Prematurity increases the risk for developing sensory processing differences, which is associated with perinatal risk factors and length of stay in the neonatal unit [42].

Our study revealed that the visual and auditory responses of neonates may be different in regard to the age of gestation. Compared to full-term infants, preterm infants have no evidence of neural changes in the occipital lobe, which, should they appear, may lead to the emergence of visual prediction [43]. Similarly, children born preterm have worse performance in auditory processing than children born full-term. Delay in sound localization is associated with deficits of the physiological mechanism of temporal processing [44]. The prematurity of all systems, especially the nervous system and sensory organs, as well as immature sensory processing, may lead to weakened responses to sensory stimuli. Furthermore, the physical environment of the hospital differs greatly from the uterine environment of a neonate’s mother from whom children were delivered prematurely [45]. Thanks to the early identification of sensory problems in preterm neonates, appropriate therapy may then be applied in earlier stages [46]. The adaptation of the physical environment, including architectural design, technical equipment, and the use of appropriate healthcare products in the hospital, is one of the corner stones that developmental care programs should apply more widely with the concept of humane neonatal care in mind [45].

## 5. Conclusions

The NBAS is an important tool that helps in assessing the neurobehavior of preterm neonates. This neurobehavioral scale is essential in the planning of appropriate therapeutic interventions. Even if decreased maturity of the ANS and habituation in preterm neonates is suspected, it is worthwhile to observe how best to support this vulnerable population. In regard to the NBAS, we can take care of these children in respect to their needs and actual condition [47]. The NBAS can help a practitioner in the assessment of the neurobehavior of preterm neonates who are at risk for developing motor and developmental disorders. The assessment of the neurobehavior of preterm neonates provides important and necessary information about the development of these infants.

The week of gestation may influence certain aspects of a child’s behavior, such as state regulation, response to sensory input, the ability to put hand to mouth, peak of excitement, cost of attention, and quality of alertness. However, taking into consideration the division between full-term and preterm neonates, there are just a few significant differences between children who were born before and after 37 weeks of gestation. The NBAS seems to indicate similar neurobehavioral outcomes for both groups of children. Even if there are no differences found between full-term and preterm infants using the NBAS assessment, this neurobehavioral scale still enables us to observe many important features of a newborn. It can help a practitioner in getting to know a preterm neonate not only as a patient in the neonatal unit but also as a person, and their particular and specific needs. This may improve the rehabilitation process and the relationship between a child and their parents, caregivers, and specialists.

## Figures and Tables

**Table 1:** Differences in assessed reflexes between premature and full-term infants (before and after 37 weeks of gestation).

Reflexes	*p* (Mann–Whitney U Test)
Ankle clonus	0.462
Sucking reflex	0.400
Glebella	0.481
Passive resist—legs	0.692
Passive resist—arms	0.787
Placing	0.326
Standing	1.000
Crawling	0.365
Incurvation	0.554
Tonic deviation of head and eyes	0.007
Nystagmus	0.349
Tonic Neck Reflex	0.122
Moro Reflex	0.465

**Table 2:** Correlation between week of gestation (without division before or after the 37th week of gestation) and the items of the NBAS.

NBAS Items	*p*	*r_s_*
Response Decrement to Tactile Stimulation of Foot	0.024	0.259
Animate visual and auditory	0.042	0.235
Peak of excitement	0.050	0.228
Cost of attention	0.041	0.236
Hand to mouth	0.387	0.101
Quality of alertness	0.010	0.292
Self-quieting	0.462	−0.086
Tremulousness	0.744	−0.038

## Data Availability

The data that support the findings of this study are available per request from the corresponding author [R.M.]. Some data are not publicly available due to containing information that could compromise the privacy of research participants.
